# Prevention and Control System of Hypokalemia in Fast Recovery After Abdominal Surgery^☆^

**DOI:** 10.1016/j.curtheres.2013.02.004

**Published:** 2013-06

**Authors:** Guanzhen Lu, Qiang Yan, Yutao Huang, Yan Zhong, Ping Shi

**Affiliations:** 1 Affiliated Central Hospital of Huzhou Teachers College, Huzhou, Zhe Jiang Province, China; 2 Nursing Department, Huzhou Teachers College, Huzhou, Zhe Jiang Province, China

**Keywords:** clinical pathway, fast recovery, gastrointestinal motility, hypokalemia

## Abstract

**Background:**

Blood potassium levels were very important during perioperative management of patients undergoing abdominal surgery. According to various worldwide studies on the causes of hypokalemia and fast-track surgeries, prehospital hypokalemia was ignored.

**Objective:**

The aim of this study to construct a prevention and control system of hypokalemia through proper clinical pathways and investigate the effects in terms of fast postoperative recovery of patients undergoing open abdominal surgery.

**Methods:**

A total of 104 patients were randomized to an observation group or a control group. The prevention and control system of hypokalemia was constructed; it was composed of 3 major modules: blood potassium monitoring, etiologic intervention, and treatment of hypokalemia. In the observation group, blood was sampled at scheduled time points (the blood potassium monitoring module) and interventions involved the preadmission and pre- and postoperative periods (etiologic intervention module). In the control group, blood sampling was delayed until after admission (blood potassium monitoring module) and interventions were only performed during the pre- and postoperative periods (etiologic intervention module). In terms of blood potassium, indices regarding gastrointestinal motility and postoperative complications were compared.

**Results:**

The severity of hypokalemia, postoperative defecation time, arrhythmia, fatigue syndrome, and urine retention differed statistically between the 2 groups (*P* < 0.05). The times to detect hypokalemia and resolve the blood condition before and after the surgery and at the first bowel sound, defecation and evacuation times differed significantly between the 2 groups (*P* < 0.01).

**Conclusions:**

The prevention and control system of hypokalemia with the starting point being before admission was more effective and allows early prevention, detection, correction, surgery, and recovery of patients undergoing open abdominal surgeries and also could be used in other specialized nursing fields.

## Introduction

Blood potassium levels could differ slightly among individuals and were very important during perioperative management of patients undergoing abdominal surgery. Abdominal surgery is a main category of general surgery, and, furthermore, the effects of postoperative potassium metabolism in patients is always a concern for physicians.^1^ The first measurement of the serum potassium level after admission to the hospital shows that many patients had had hypokalemia before, which could not be explained by common causes such as inadequate intake or excessive loss of potassium. With the development of economy, improvement of living standards, increase in work pressure, and changes in lifestyle, the primary disease spectrum has altered greatly, resulting in hypertension and diabetes mellitus (DM) becoming very common conditions.^2,3^ Medications, health-care products, and concomitant lifestyle factors have some effects on the distribution and shifting of potassium within the body but were never paid much attention. Moreover, digestive organs were primarily involved in abdominal surgeries, and diet was closely related with differences in blood potassium levels. All of the aforementioned made the causes of hypokalemia after hospital admission more complex. In clinical practice, surgery delay due to hypokalemia was a common occurrence, and even mild hypokalemia was a contraindication to for surgery. Management of hypokalemia during the perioperative period was too late. Hypokalemia occurred for many different controlled and uncontrolled reasons. In addition, there were still some controlled causes that could be prevented through nursing intervention methods. According to various worldwide studies on the causes of hypokalemia and fast track surgery options,^1,4^ prehospital hypokalemia was ignored. In this study, the prevention and control system of hypokalemia was constructed using 3 optimal major modules, and a fast recovery was achieved through a wide range of interventions (eg, both in and out of the hospital) for patients undergoing open abdominal surgeries.

## Methods

### Settings and patients

A total of 104 patients were enrolled in this study. All patients underwent selective caesarean operation at our hospital and presented with hypokalemia from June 2009 to August 2011. All patients were randomized to an observation group (n = 50, 28 males and 22 females) with a mean (SD) age of 47.16 (17.34) years (range, 16–70 years) and a control group (n = 54, 31 males and 23 females) with a mean (SD) age of 46.51 (16.82) years (range, 17–69 years). In the observation group, 18 patients underwent cholecystectomy plus biliary drainage, 16 patients underwent subtotal gastrectomy, 10 underwent colorectal cancer surgery, 3 underwent splenectomy, and 3 underwent retroperitoneal tumorectomy. In the control group, 20 patients underwent cholecystectomy plus biliary drainage, 17 patients underwent subtotal gastrectomy, 11 underwent colorectal cancer surgery, 4 underwent splenectomy, and 4 underwent retroperitoneal tumorectomy. The 2 groups were comparable in terms of the general data, occupation, education level, prehospital hypokalemia severity, method of anesthesia, surgical procedure, and catheter type (*P* > 0.05).

Inclusion criteria were blood potassium levels <3.5 mmol/L at the first testing in the outpatient department, selective types 3 and 4 open abdominal surgery, successful general anesthesia, and stable vital signs 6 hours postoperatively. Patients with an acute abdomen, history of abdominal surgeries, major organ dysfunction, or basic diseases related with hypokalemia were excluded from this study.

### Criteria for hypokalemia

Electrolytes were measured using the 7600 biochemical analyzer (Hitachi, Tokyo, Japan) with the ion electrolysis method. Normal serum potassium levels were between 3.5 and 5.5 mmol/L, and a level <3.5 mmol/L was defined as hypokalemia.^5^ Slight, moderate, and severe hypokalemia was in the range of 3.0 to 3.5 mmol/L, 2.5 to 3.0 mmol/L, and <2.5 mmol/L levels, respectively.^6^

### Interventions

#### Observation group

Outpatients who chose selective open abdominal surgeries were informed about the study’s objective, method, and significance. The volunteers for this study were provided with the necessary informed consent forms. Participants’ electrolyte levels were measured in the outpatient department initially. Patients with low potassium levels were eventually recruited in this group according to a random numbers table. Nurses notified patients both verbally and in writing about the necessary preparation before their admission to the hospital, with an emphasis on the etiologic intervention of hypokalemia. All patients were instructed to fast the night before hospital admission. On admission, blood electrolytes, hepatic and renal function, glucose, and fat were measured. Blood potassium was monitored after admission. Blood sampling was performed using 4 conventional time points: on admission, 12 hours preoperatively, and 24 and 48 hours postoperatively. Samples were analyzed, and results were acquired in a timely manner. The etiologic intervention started before admission to the hospital.

#### Control group

Patients were informed verbally about the routine preparation before being admitted to the hospital. Patients undergoing no etiologic intervention for hypokalemia were included in this group. Blood was collected on an empty stomach according to the fasting time, conventionally in 2 hours postprandially, 12 hours preoperatively, and 24 and 48 hours postoperatively. The first blood sampling time was delayed than in the observation group, and the etiologic intervention started after hospital admission.

Hypokalemia was treated according to conventional treatment process in both groups.

### Prevention and control system framework of hypokalemia

**Figure 1:**
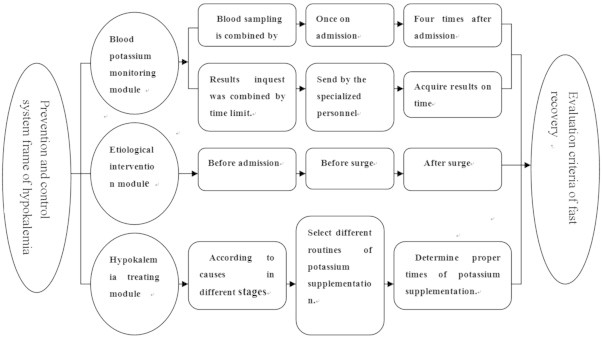
Prevention and control system framework of hypokalemia.

The prevention and control system framework of hypokalemia was composed of 3 major modules: the blood potassium monitoring module, etiologic intervention module, and hypokalemia treatment module. This simple framework is shown in Figure 1.

### Evaluation indices

#### Observation indices of blood potassium

The observation indices of blood potassium included (1) the time to detect hypokalemia, defined as the time from hospital admission to diagnosis of hypokalemia by laboratory test results, and (2) the time to resolution of hypokalemia, defined as the time from diagnosis to resolution of hypokalemia.

#### Observation indices of gastrointestinal motility

Observation indices of gastrointestinal motility included (1) the first bowel sound time, defined as the time from the completion of the surgery to the first bowel sound; (2) the first evacuation time, defined as the time from the completion of surgery to the evacuation of anus returning; and (3) the first defection time, defined as the time from the completion of surgery to the first spontaneous defecation after the surgery.

### Postoperative complications

Enteroparalysis, arrhythmia, wound dehiscence and infection, urine retention, and postoperative fatigue syndrome after surgery were observed. They were directly related with the fast postoperative recovery and hospitalization period.

### Statistical analysis

SPSS version 17.0 (SPSS Inc, Chicago, Illinois) was used for statistical analysis. All data were expressed as the mean (SD). Numeration data were analyzed with the χ^2^ test, and measurement data were analyzed with the Student *t* test. Ranked data were analyzed using the Wilcoxon rank sum test.

## Results

Through the whole intervention in and out of the hospital, the degree of low potassium before surgery and the mean time to correct the disorder after surgery in the observation group were less than in the control group (*P* < 0.05) (Table I). The mean time to detect hypokalemia and correct the disorder after surgery in the observation group were also less than in the control group (*P* < 0.01) (Table I).

Table II indicated that all of the first defecation times (*P* < 0.05), the first bowel sound and evacuation times after surgery (*P* < 0.01) in the observation group were shorter significantly.

Moreover, Table III showed that postoperative electrocardiogram abnormalities, fatigue syndrome, and urine retention were milder in the observation group than in the control group (*P* < 0.05) and that the mean number of days of hospitalization were fewer in the observation group than in the control group (*P* < 0.01).

## Discussion

### Causes and significance of preventing and controlling hypokalemia

Potassium is the most important inorganic cation with total body potassium stores of 50 to 55 mmol/kg. Ninety percent of the body’s potassium is intracellular, and only 1.4% is in the extracellular fluid. Only when the intake and discharge of potassium are in dynamic equilibrium can the serum level remain in the normal range. Hypokalemia is caused by inadequate intake or loss of potassium and can occur for many reasons. Abdominal surgery is characterized by more tissues involved in, severe damage, much exudation in the surgical field, excessive loss of digestive fluid, and long fasting times, as well as an inhibition of gastrointestinal mortality after the surgery. Furthermore, electrolyte disturbance, especially hypokalemia, is one of the most common complications of major abdominal surgery. Posttraumatic hypokalemia is related to the severity of the trauma.^7^

Vanek VW et al had been interested in postoperative potassium mechanism in the 1990s.^8^ Chinese researchers determined that intravenous potassium supplementation in the early stage after abdominal surgery is beneficial for the recovery of gastrointestinal function. Internal Chinese reports proved that postoperative blood potassium levels were positively correlated with the preoperative levels in patients undergoing elective gynecologic surgery. The aforementioned studies were limited to the perioperative period. However, clinical observations showed that hypokalemia that was found on admission to the hospital actually existed before admission.

A prehospital study showed that the time of elective surgery was shortened after correcting hypokalemia. Therefore, we constructed a hypokalemia prevention and control system and introduced this system to the fast track recovery of abdominal surgery, which had not been reviewed in PubMed, the China Hospital Knowledge Database, and WanFang Digital Database. Therefore, this study is of academic value. Thus, the following control factors should be controlled as early as possible before admission to the hospital. Eating a diet high in potassium is recommended. Diet is the safest way to supplement potassium. Potassium intake can effectively reduce the systolic pressure and diastolic pressure in patients with hypertension as well as healthy adults.^9^ Good sources of potassium include, but are not limited to, fruits such as bananas and oranges. Furthermore, blood pressure responds to a low-sodium and high-potassium diet in a direct and progressive manner, regardless of sex and race,^2^ and to a balance between work and leisure. High-intensity labor should be avoided, especially in the summer, because excessive perspiration increases potassium loss. Proper exercise increases the blood potassium. Balanced meals are encouraged. Eating and drinking too much at 1 meal or high sweet food should be avoided, especially when overtired, because excessive glucose could shift the potassium into the cells. Similar effects also occur in diabetic patients.^10^ Ensure proper use of medications. In this study, 8 patients lost potassium due to long-term use of diuretics,^5^ 4 patients took antibodies, which altered the transcellular potential difference and easily moved the potassium from cells, and 4 patients used antacid agents for acidosis. Potassium is related to the regulation of the acid-base balance in the body and an increase of 0.1 in the pH corresponds to a shift of 0.1 to 1.0 mmol/L potassium into the cells. Drug-induced hypokalemia was reported.^11–13^ Consequently, drugs for the treatment of primary diseases should be screened for side effects to prevent drug-induced hypokalemia. The potential for hypokalemia should be evaluated. Conditional sampling was used for blood in the outpatient department to rule out the possibility of asymptomatic hypokalemia. An oral supplement can modulate it directly, and mild hypokalemia was generally corrected.

Comprehensive prevention and management of hypokalemia often had important clinical significance: (1) they relieve the shortage of beds and shorten the surgical waiting period; (2) they allow for early prevention, discovery, correction, surgery, and recovery for hypokalemia patients undergoing open abdominal surgeries^1,8^ ; (3) they predict the risk of hypokalemia in the various stages through inducements; (4) they integrate medical treatment, nursing, prevention, and education; and (5) clinical pathways are advocated, which was the necessary trend of the nursing field and provides experiences for the application in other departments.

### Advantages of the prevention and control system for hypokalemia

#### A reasonable clinical pathway is beneficial for the early prevention, discovery, correction, surgery, and recovery of this potassium-deficient condition

De Bleser et al^14^ first presumed^]^ that a clinical pathway was a way to manage a patient's treatment process. A clinical pathway was structured with proper multidisciplinary plans that are sequential with a medical timeline that was within the realm of medical and nursing practice for treating specified diseases or when performing surgical procedures to reduce the delayed recovery of the aforementioned patients. Furthermore, this pathway was structured to decrease the delay and waste of medical resources and optimize the quality of patient care. All procedures of every module in this study were performed at sequential time points, which caused intervention measures to normalize and be combined by time, which therefore ensured the implementation of every procedure. In the observation group, hypokalemia was considered to have existed before hospital admission, and blood was sampled on and after admission to the hospital, in accordance with the blood potassium monitoring pathway, which comprehensively reflects the dynamic changes in blood potassium before admission and pre- and postoperatively. In the control group, hypokalemia was not diagnosed by etiologic factors before admission to the hospital, and blood potassium levels were measured in the fasting blood sampled routinely, which delayed the diagnosis and treatment of hypokalemia. Table I shows that the severity of low potassium differed statistically between the 2 groups (*P* < 0.05) and the mean times to detect hypokalemia and correct the disorder after surgery also differed significantly between the 2 groups (*P* < 0.01), which suggested that the intervention method in the observation group decreased the severity of hypokalemia and gained the treatment time due to early detection. This pathway took full advantage of early prevention, detection, and treatment, laying a foundation for early surgery and rehabilitation.

#### A comprehensively constructed system can promote fast recovery after surgery

In recent years, a new concept of "fast rehabilitation" was introduced in the surgical field to use a series of optimal, evidence-based intervention measures in the perioperative period to reduce mental and physical traumatic stress and achieve the goal of fast rehabilitation.^4^ Most open abdominal surgeries are performed under general anesthesia. These patients are susceptible to abdominal distention, enteroparalysis, accelerated heart rate, increased oxygen consumption, and hypopnea, all of which are termed “postoperative fatigue syndrome.”^15^ This syndrome greatly endangers the patient. Therefore, elimination of abdominal distention and recovery of evacuation in the early stages after the surgery is very important for the prevention of postoperative complications. Table II indicated that the time to the first defecation after the surgery differed statistically between the 2 groups (*P* < 0.05) and the first bowel sound and evacuation times after surgery differed significantly between the 2 groups (*P* < 0.01), illustrating that the intervention method in the observation group can relieve the inhibition of gastrointestinal motility at an early stage postoperatively and gain time for oral potassium supplementation. This was in line with the results of Franse LV,^16^ in that there was no significant difference between intake of potassium-rich foods and oral potassium for postoperative patients.

For patients undergoing open abdominal surgeries, enterokinesia completely disappears in the first 12 to 24 hours postoperatively. Temporary enteroparalysis is the most common postoperative motility disorder and occurs in nearly all patients undergoing major abdominal surgeries. Delayed enteroparalysis may develop in 15% of patients, which was one of the important factors that led to a patient’s hospitalization. Hypokalemia is critical in the delay of gastrointestinal function recovery. Furthermore, hypogastric nerves, surgical dragging, and structure shift also may induce changes in bladder motility after the surgery, in addition to a temporary urination disorder that may occur after removing the urinary catheter. Table III shows that the postoperative electrocardiogram abnormalities, fatigue syndrome, and urine retention were milder in the observation group than in the control group (*P* < 0.05) and that the mean number of days of hospitalization was less in the observation group than in the control group (*P* < 0.01), which may be due to the slightly lower potassium levels, early detection, and correction in the observation group. In both groups, the high incidence of enteroparalysis confirmed the possibility of enteroparalysis after major abdominal surgery, although the low incidence of wound infection or dehiscence suggested that enteroparalysis was generally temporary because severe and persistent enteroparalysis could induce the release of inflammatory mediators and obvious abdominal bulge, easily causing wound infection or dehiscence. These created favorable conditions for a fast recovery.

### Suggested method and characteristics

In this study, the prevention and control system of hypokalemia was optimally constructed according to a reasonable clinical pathway. The basic framework was composed of 3 major modules. The first was the monitoring of blood potassium module, which was the principal line of the system that samples blood at time points and acquires results in a specific time period. In addition to in the outpatient department and 4 routine time points after admission, blood could be sampled within other random time points conditionally to guarantee the continuity of blood potassium monitoring. The electrolyte results would be reported 2 hours after sending the blood samples to the hospital laboratory, which delays the time for scientific potassium supplementation correctly. We sent samples and acquired results at strictly scheduled time points (within 60 minutes after sending) in the hospital information system by the specialized personnel, which was more effective in terms of supplying potassium for physicians. The second was the etiologic intervention module, which was the basis of this system. The etiologic intervention module involved the whole range (eg, preadmission to the hospital and pre- and postoperatively). In the outpatient department, patients first received postoperative instructions and with preventive hypokalemia medications. This was an innovative measure of optimal flow management and breaks the pattern that the process is confined to the hospital. The third was the hypokalemia-treating module, which was the key to fast recovery postoperatively. It had been proved that early intravenous potassium supplementation after the abdominal surgery was beneficial for the recovery of gastrointestinal function. The preoperative serum potassium level was lower than that in preoperative in nondigestive surgeries. Because all patients in our study were hypokalemic, we paid more attention to the time of postoperative potassium supplementation and strictly followed the principle of supplying the potassium at the stage of urine discharge. The potassium could be administered 1 to 2 hours postoperatively when the urine volume was ∼20 mL/h, and in this study, the potassium-supplying time (1–2 hours postoperatively) was earlier than previously (1 day postoperatively) and was also a new strategy in the treatment of hypokalemia. Certainly, false anuria should be ruled out. How and what to supply were determined according the blood potassium levels, symptoms, signs, and electrocardiogram.

## Conclusions

In this study, a prevention and control system of hypokalemia integrating medical treatment, nursing, prevention, and education was constructed through an optimal combination of the intervention treatment flow of hypokalemia. This method was simple and practicable and could not only achieve the goals of early prevention, detection, correction, surgery, and recovery, but also could be beneficial for the increase of judgment ability of young nurses and students. This method introduced a new concept to surgical nursing. The disadvantage of this study was not to perform the intragroup comparison of the blood potassium levels at the conventional blood-sampling time points. Further investigation should be done to improve this system.

## Conflicts of Interest

The authors have indicated that they have no conflicts of interest regarding the content of this article.

## Figures and Tables

**Table I:** Comparison of indices of blood potassium between the 2 groups.

		Severity of Hypokalemia Before Admission (No. of Patients)				Severity of Hypokalemia After Admission (No. of Patients)				Before Surgery, h, Mean (SD)		After Surgery, h, Mean (SD)
Group	n	Normal	Mild	Moderate	Severe	Normal	Mild	Moderate	Severe	Time to Detection of Hypokalemia	Time to Resolution of Hypokalemia	Time to Resolution of Hypokalemia

Observation	50	0	46	4	0	19	30	1	0	2.92 (0.84)	16.98 (4.37)	25.36 (6.51)
Control	54	0	50	3	1	2	41	9	2	9.47 (2.86)	20.82 (5.64)	35.42 (8.28)
t/u value		u = 0.11				u = 6.78			t = 16.10		t = 3.90	t = 6.91
*P*		>0.05				<0.05			<0.01		<0.05	<0.01

**Table II:** Comparison of indices of gastrointestinal motility after the surgery, h, mean (SD).

Groups	N	Time to First Bowel Sound	Time to First Evacuation	Time to First Defecation	Time to First Micturition
Observation	50	42.79 (9.48)	58.43 (11.98)	94.45 (19.36)	5.62 (1.64)
Control	54	49.14 (11.05)	70.21 (13.57)	104.21 (20.73)	6.36 (1.71)
*t* test value		3.15	4.70	2.48	−2.25
*P*		<0.01	<0.01	<0.05	<0.05

**Table III:** Comparison of postoperative complications.

		Postoperative Complications (No. of Patients)					
Group	N	Enteroparalysis	Arrhythmia	Wound Dehiscence and Infection	Urine Retention	Postoperative Fatigue Syndrome	Mean (SD) Length of Hospitalization, d

Observation	50	40	8	4	2	2	13.78 (4.37)
Control	54	46	19	13	5	10	17.24 (6.53)
t/χ^2^ value		x^2^ = 0.19	x^2^ = 4.02	x^2^ = 3.80	x^2^ = 0.46	x^2^ = 4.03	t = 3.20
*P*		>0.05	<0.05	<0.05	>0.05	<0.05	<0.01
